# Resurgent anthrax in Bangladesh: Operationalizing the Lancet One Health Commission framework for endemic preparedness

**DOI:** 10.1016/j.onehlt.2026.101427

**Published:** 2026-04-24

**Authors:** Md. Saiful Islam, Andrea Sylvia Winkler, John Humphrey Amuasi, Xiang Yang, Md. Tanvir Rahman

**Affiliations:** a Department of Animal Science, University of California – Davis, Davis 95616, United States; b Department of Microbiology and Hygiene, Faculty of Veterinary Science, Bangladesh Agricultural University, Mymensingh 2202, Bangladesh; c Department of Neurology, TUM University Hospital, and Center for Global Health, School of Medicine and Health, Technical University of Munich (TUM), Munich, Germany; d Department of Community Medicine and Global Health, Institute of Health and Society, University of Oslo, Oslo, Norway; e Department of Global Health, Kwame Nkrumah University of Science and Technology, Kumasi, Ghana; f Bernhard Nocht Institute of Tropical Medicine, Hamburg, Germany

Anthrax has persisted as an important zoonotic disease despite advances in biomedical research, owing to several complex socioecological factors. *Bacillus anthracis* spores can survive, particularly in soil, for far in excess of 60 years, or even hundreds of years, creating persistent environmental reservoirs that can trigger outbreaks in human and animal populations (Fig. 1) [1]. This opinion argues that operationalizing the principles of the Lancet One Health Commission (LOHC) [2] is essential to address the persistent endemicity of anthrax in Bangladesh.

**Fig. 1:**
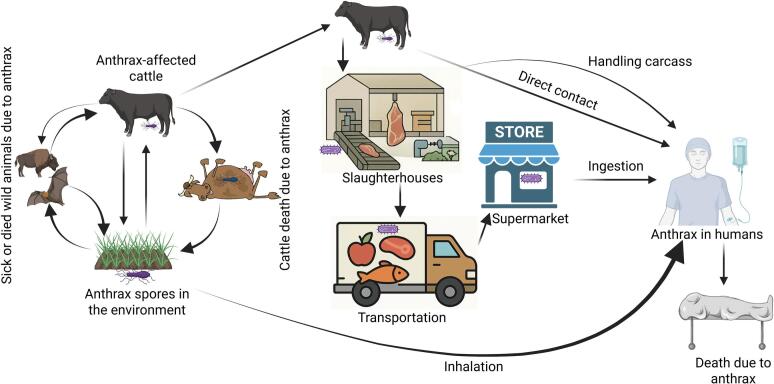
Possible transmission pathways of anthrax involving humans, other animals, and the environment (prepared in https://BioRender.com).

Anthrax is highly fatal if untreated. The most common human anthrax infection occurs in cutaneous form, while the gastrointestinal anthrax, resulting from ingestion of contaminated meat, can cause severe gastrointestinal illness. Inhalational anthrax, another form of anthrax, is rare but becomes the deadliest if left untreated [3]. In July and September 2025, there was an outbreak of anthrax in the Rangpur division of Bangladesh, resulting in 17 confirmed human cases and two deaths. The deceased were a 38-year-old male and a 48-year-old female; both had skin lesions typical of anthrax [4]. Though livestock deaths, several later confirmed as anthrax, had been reported weeks earlier, mass vaccination of cattle was administered only after human infections had already spread [5]. This highlights the recurring themes of delayed diagnosis, vaccination in response to infection, and limited coordination between human and animal health services, which were further exacerbated by the centralized diagnostic capacity in national laboratories, often resulting in slow confirmation and gaps in real-time surveillance. These factors suggest that anthrax outbreaks are likely a consequence of systemic failures in prevention and preparedness.

Bangladesh has been battling anthrax for many years. Human anthrax has been repeatedly documented in Bangladesh, with 27 suspected cases reported during 1980–1984, 1857 cases during 2009–2015, 2186 cases during 2016–2020, 607 cases and two deaths during the 2010 epidemic alone, and 1119 suspected cases reported during 2022–2023 [5], [6], [7], [8]. Importantly, previous reports indicate that anthrax outbreaks in Bangladesh tend to occur in certain seasons and may be linked to environmental factors such as rainfall, flooding, and soil disturbance [9]. These ecological conditions can facilitate the re-emergence of *B. anthracis* spores from environmental reservoirs and increase the risk of exposure among grazing livestock. Taking such environmental factors into account, surveillance systems could improve early detection and prevention strategies within an integrated One Health framework. Animal anthrax reflects a similar incidence rate, with thousands of cases recorded since the 1980s [10]. The Institute of Epidemiology, Disease Control, and Research (IEDCR) has been improving the surveillance and reporting system, but the frequency of outbreaks appears to remain high [11]. The 2025 outbreak in Rangpur is an example of the continuance of the deep-rooted problem structurally. The mass animal vaccination campaigns were initiated only after the occurrence of human diseases, hospitals relied on central laboratories due to a shortage of diagnostic capacity, and the veterinary services primarily focused on vaccinating cattle. Moreover, public health services, primarily upazila health complexes and district hospitals, managed human patients with little coordination with veterinary and environmental health services because of the absence of established One Health protocols. Meanwhile, rural households suffering from economic hardship mostly still sold or slaughtered sick animals for consumption, which introduced contaminated meat into the food supply chain and perpetuated the anthrax transmission cycle [5]. These patterns reflect what LOHC highlighted, namely, governance being fragmented, integration being weak, and operational clarity of One Health practice being insufficient [2].

Reframing the concept of anthrax from the LOHC perspective highlights that the disease should be considered not only a veterinary or public health concern, but also a socioecological challenge. The Commission's ethos, based on holism, systems thinking, equity, stewardship, and epistemological pluralism, provides an interpretive framework that makes visible the interdependencies that should be considered in designing and implementing anthrax control [2]. Holism and systems thinking emphasize the need to link livestock and human vaccination, human diagnosis and treatment, food safety oversight, and soil ecology. This is important because carcass disposal and flooding events can perpetually re-contaminate environmental reservoirs, such as soil and water bodies, which serve as long-term reservoirs for *B. anthracis* spores. Equity reminds us that smallholder farmers are affected heavily during outbreaks. They often lack the resources for preventive vaccination and suffer greater socioeconomic losses due to animal deaths. Stewardship emphasizes that governments should proactively invest in prevention systems, including annual mass livestock vaccination programs, safe carcass disposal systems, and surveillance systems that remain operational during climate-related disruptions, such as flooding or extreme weather. Epistemological pluralism requires incorporating community knowledge and economic realities into control measures. Awareness campaigns that fail to align with the local situation often lack effectiveness in changing risky behaviors, such as slaughtering sick animals and selling contaminated meat.

The “future trajectories” commentary from the LOHC further indicates that zoonotic threats such as anthrax should be part of broader One Health agendas [12]. Incorporating anthrax into endemic and outbreak preparedness frameworks would enhance early detection and vaccine readiness, thereby averting significant socioeconomic losses. This ensures that control of endemic zoonoses is not overlooked in favor of larger pandemic threats. Integrated surveillance that connects veterinary, environmental, and clinical data, possibly with the support of artificial intelligence tools, would enable real-time mapping of hotspots. The rapid urbanization in Bangladesh may increase the connectivity among livestock movements in peri-urban areas, which may create new exposure pathways and highlight the need to integrate anthrax into urban health surveillance. Equity and epistemological pluralism should be the main focus. It is important to engage women and other marginalized groups, such as landless farmers, smallholder livestock keepers, and ethnic minority communities, in animal care when designing community outreach and prevention strategies [13]. Moreover, integrating Information, Education, and Communication (IEC) and Behavior Change Communication (BCC) activities can further facilitate the translation of scientific and indigenous knowledge into locally appropriate practices through interactive, community-based engagement, using tailored messages and multiple communication channels to promote and sustain positive behavioral change [14]. Migration and mobility of livestock and humans within Bangladesh alter exposure routes, so anthrax preparedness should be incorporated into broader food security and displacement planning. Tools for metrics and accountability, such as the Global One Health Index, could help in measuring progress in anthrax control [15]. Sustainability incorporates preventive vaccination and carcass management into regular livestock extension services rather than relying only on episodic emergency campaigns.

Linking these One Health prevention and response frameworks to the Bangladesh scenario, it becomes evident that zoonotic disease, anthrax, is a viable test case for implementing the One Health concept in areas with limited resources and high disease burden [16]. The disease's established ecology, regular outbreak, and socio-economic disruption reveal the weakness of fragmented governance and reactive campaigns. Bangladesh should, therefore, institutionalize preventive animal vaccination, move adequate modern diagnostics to district hospitals and veterinary laboratories, integrate surveillance across human, animal, and environmental sectors, and integrate One Health into governance at the ministerial level. It is crucial to build community partnerships through trusted leaders. Health interventions should be connected to economic incentives and local knowledge systems.

The 2025 Rangpur outbreak is more than a localized crisis; it reflects a broader problem of endemic zoonotic diseases worldwide. Ignoring anthrax while only focusing on pandemic preparedness could leave millions at risk for repeated outbreaks. They can disrupt food systems, worsen social inequities, and damage trust in public health. Yet the repeated emergence of anthrax also presents an opportunity to rethink how prevention and control are implemented. By applying the LOHC values and future trajectories, Bangladesh can show how One Health can be made practical, operational, and sustainable. Breaking the cycle of anthrax transmission with systemic, equity-focused, and multi-sectoral interventions would reduce the impact of this neglected zoonosis. It would be resilient against a wide range of emerging infectious diseases. Therefore, in a way, anthrax is not only a reminder of an ancient pathogen's persistence, but also a lens through which the urgency of One Health becomes undeniable.

## CRediT authorship contribution statement

**Md. Saiful Islam:** Writing – review & editing, Writing – original draft, Visualization, Methodology, Investigation, Data curation, Conceptualization. **Andrea Sylvia Winkler:** Writing – review & editing, Validation, Supervision, Conceptualization. **John Humphrey Amuasi:** Writing – review & editing, Validation, Supervision, Conceptualization. **Xiang Yang:** Writing – review & editing, Validation, Supervision, Conceptualization. **Md. Tanvir Rahman:** Writing – review & editing, Validation, Supervision, Resources, Project administration, Conceptualization.

## Funding

No funding was received.

## Declaration of competing interest

Authors declare no conflicts of interest.

## Data Availability

No data was used for the research described in the article.
